# Structural biology in the age of X-ray free-electron lasers and exascale computing

**DOI:** 10.1016/j.sbi.2024.102808

**Published:** 2024-03-27

**Authors:** Sandra Mous, Frédéric Poitevin, Mark S. Hunter, Dilipkumar N. Asthagiri, Thomas L. Beck

**Affiliations:** 1 Linac Coherent Light Source, SLAC National Accelerator Laboratory, Menlo Park, 94025, CA, USA; 2 National Center for Computational Sciences, Oak Ridge National Laboratory, Oak Ridge, 37830-6012, TN, USA

**Keywords:** Conformational dynamics, Biomolecules, Metalloproteins, Nucleic acids, Forcefields

## Abstract

Serial femtosecond X-ray crystallography has emerged as a powerful method for investigating biomolecular structure and dynamics. With the new generation of X-ray free-electron lasers, which generate ultrabright X-ray pulses at megahertz repetition rates, we can now rapidly probe ultrafast conformational changes and charge movement in biomolecules. Over the last year, another innovation has been the deployment of Frontier, the world’s first exascale supercomputer. Synergizing extremely high repetition rate X-ray light sources and exascale computing has the potential to accelerate discovery in biomolecular sciences. Here we outline our perspective on each of these remarkable innovations individually, and the opportunities and challenges in yoking them within an integrated research infrastructure.

## Introduction

The key to understanding proteins, nucleic acids, and other molecules of life depends on knowing the atomic arrangements in these molecules and how those arrangements change in response to interactions with the solvent or other molecules. To a great extent, our understanding of bio-macromolecular structure and function has relied on studying single, static structures, complemented by molecular simulations in favorable cases. Recent developments in time-resolved structural biology methods and molecular simulations promise to build on our knowledge of these static structures with a wealth of dynamic information, leading to a better understanding of the relationship between structure, dynamics, and function. In particular, the emergence of extremely bright X-ray light sources and exascale computing promises to usher in a new era of *dynamic* structural biology.

## Studying the structural dynamics of biomolecules using time-resolved crystallography

**Figure 1:**
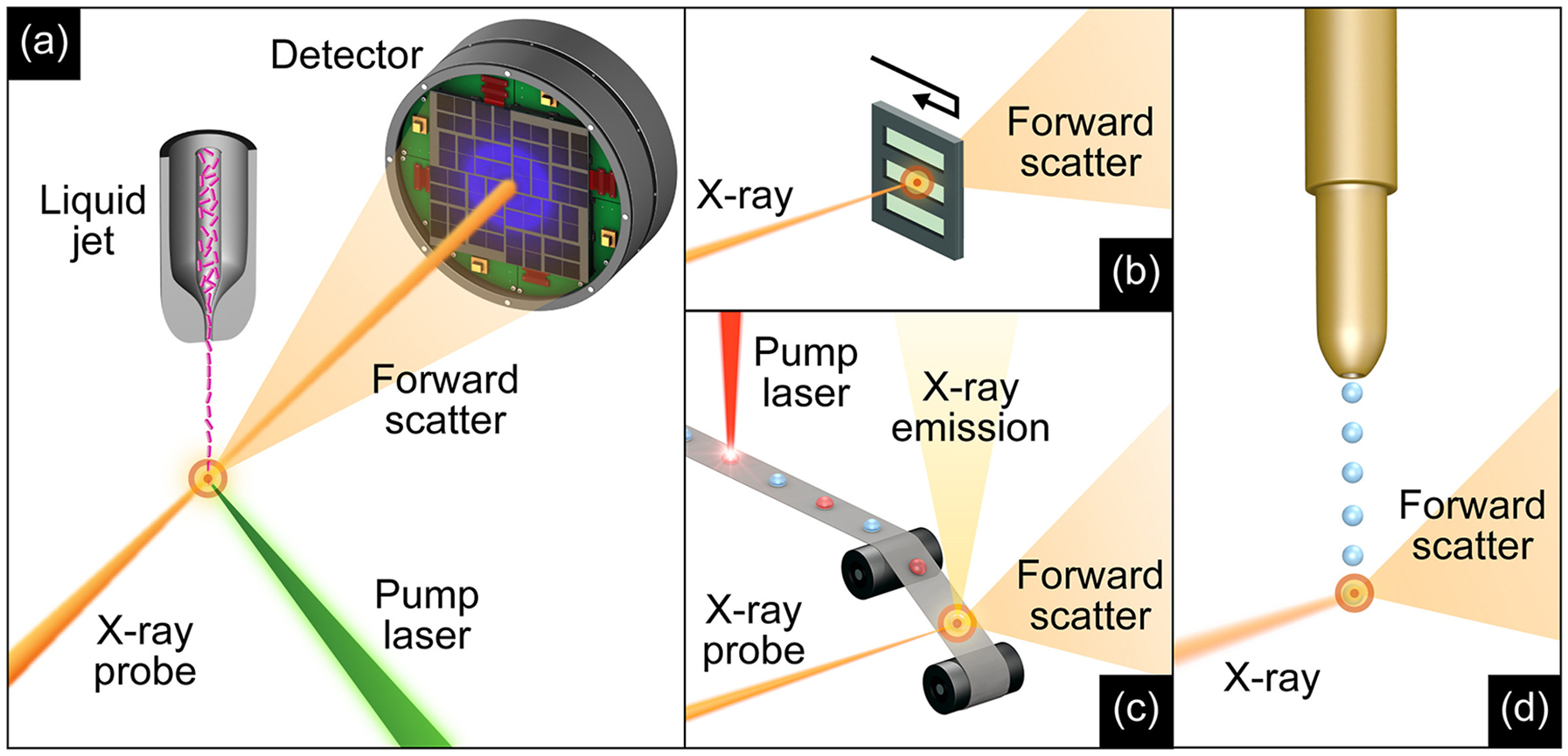
**(a)** Schematic illustrating the serial femtosecond X-ray crystallography (SFX) experiment. The liquid jet replenishes the crystalline sample at the interaction point, where the sample interacts with the X-ray pulse. Forward scattering data, which encodes the structural information, is collected by a detector placed close to the interaction point. By pumping the sample with an optical laser pulse shortly before the X-ray pulse arrives, the dynamics of photosensitive systems can be studied. **(b)** Fixed-target sample delivery methods bring the crystalline sample into the interaction point using a chip or other sample holder [16]. **(c)** Tape-drive systems use piezo-acoustic injectors to deposit sample droplets on a conveyor belt system, from which X-ray diffraction and emission data can be collected simultaneously [17]. **(d)** Automated droplet-on-demand systems are another piezo-injector-based sample delivery system [18].

X-ray free-electron Lasers (XFELs), such as the Linac Coherent Light Source (LCLS) [1], have opened a new window to study the structural dynamics of macromolecules using serial femtosecond X-ray crystallography (SFX). XFELs produce X-ray radiation with exceptional properties, offering a billion-fold increase in peak brightness compared to synchrotron radiation. The femtosecond duration of the XFEL pulses ensures that atomic-level structural information is captured before the ionizing radiation can induce secondary electronic and atomic rearrangements (often referred to as “diffraction-before-destruction”) [2]. As a result, performing measurements on samples at cryogenic temperatures – typically used for studying crystals at synchrotron light sources and in modern electron microscopy methods to mitigate radiation damage – is no longer necessary. Structures of macromolecules can therefore be obtained at near-physiological temperatures, including structures of particularly radiation-sensitive samples, including metalloenzymes [3–8] and protein radicals [9]. While the femtosecond pulse duration removes the need for cryogenic cooling of the samples, the high peak brightness of XFEL radiation enables X-ray diffraction data to be collected from crystals that are several micrometers or smaller in size. This capability has proven beneficial for the structural study of membrane proteins such as the medically relevant G-protein coupled receptors [10–13]. However, the high peak brightness of the X-ray pulse also damages the sample shortly after the diffraction data are obtained, and therefore samples must be continually replenished in the X-ray interaction region (Figure 1) [14,15].

At XFELs, dynamics can be probed by expanding the experimental setup with a trigger, such as an optical pump laser (Figure 1a) [19] or chemical pump, using a mixing device [20]. By varying the time delay between the trigger and the X-ray pulse, macromolecular dynamics can be captured with femtosecond time resolution. Time-resolved methods at the XFEL have greatly increased our understanding of the ultrafast chemistry occurring within photosensitive systems (e.g., photoactive yellow protein [21], photosystem II [22], and the visual pigment rhodopsin [23]) and have captured the structural changes that occur in macromolecules upon ligand binding (including e.g., RNA [24,25] and proteins that produce or provide resistance to antibiotics [3,26]). Recently, the application area of time-resolved studies at the XFEL has been broadened through the development of new triggering methods, such as photocaged compounds [27], temperature jump excitation [28], and electric field stimulation [29].

Recent years have seen an increase in multimodal experiments, which help to validate X-ray diffraction studies through optical spectroscopy and computational simulations [30–33]. It is also possible to capture X-ray diffraction and X-ray spectroscopy data simultaneously (Figure 1c), such that changes in both the atomic and electronic structure can be followed in time [34]. Among other examples, such multimodal experiments have recently enabled researchers to monitor the iron oxidation state while capturing the conformational changes of isopenicillin N synthase during catalysis [3]. New X-ray methods are still being developed, further expanding the toolkit for multimodal studies of macromolecular dynamics. For example, XES in the tender X-ray regime (~1.5–5 keV) provides an exciting opportunity to capture the electronic state of elements common in biological systems, including sulfur, phosphorus, chlorine, and calcium [35]. The tender X-ray energy regime will also enable K-edge anomalous scattering methods [36] to be developed.

**Figure 2:**
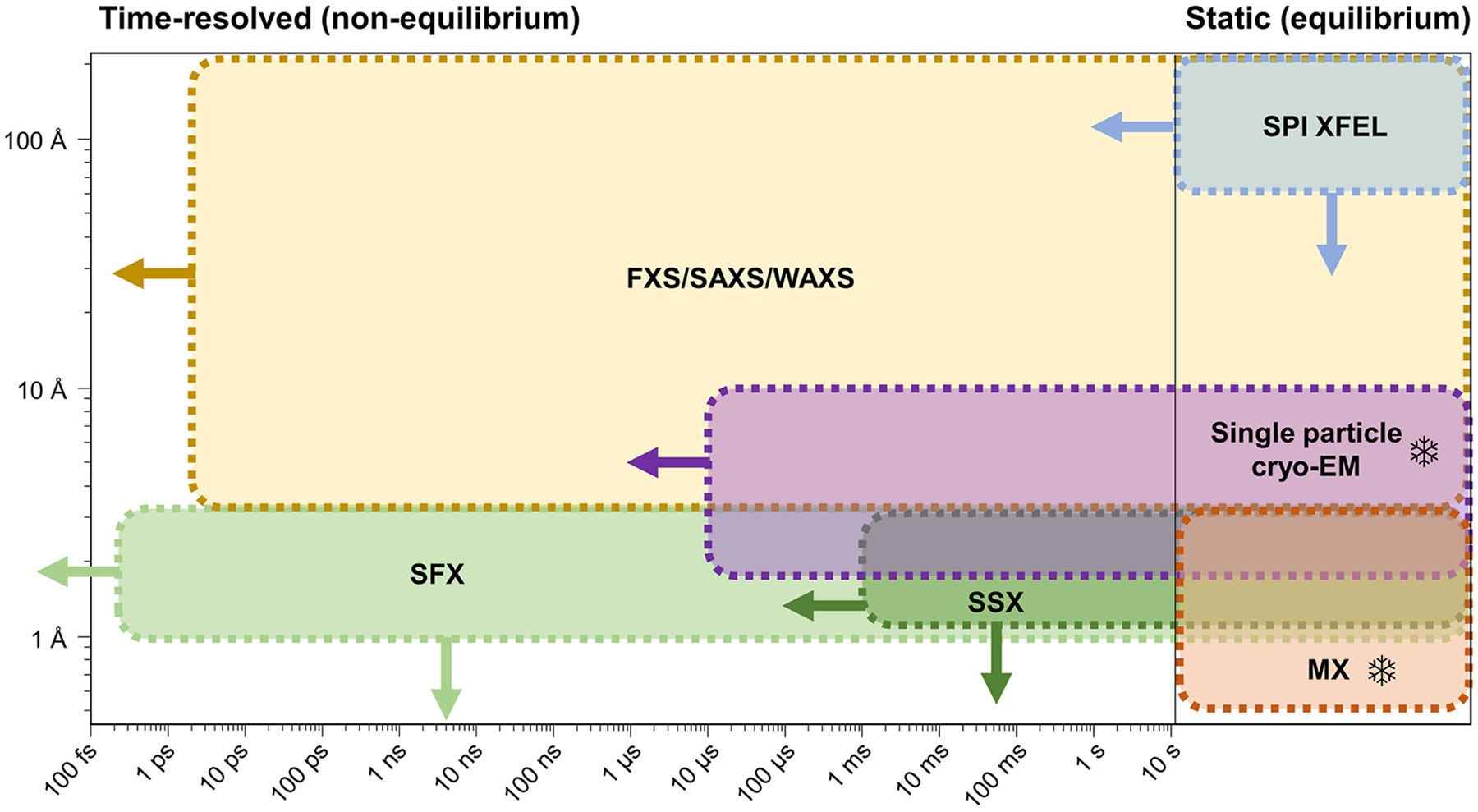
Resolution ranges achieved by the different diffraction and scattering techniques used for determining static structures and time-resolved dynamics: fluctuation X-ray scattering (FXS), small/wide-angle X-ray scattering (SAXS/WAXS), single particle imaging (SPI), cryogenic electron microscopy (cryo-EM), serial femtosecond X-ray crystallography (SFX), serial synchrotron X-ray crystallography (SSX), and macromolecular X-ray crystallography (MX). Methods that rely on cryogenic cooling of the sample (cryo-EM and MX) are marked with an ice crystal. Arrows indicate the expected improvement in resolution driven by further method development. It should be noted that the resolution range for SPI is expected to quickly expand in the following years as MHz XFELs become available.

Further complementary methods exist to probe biomolecular structure and dynamics (Figure 2). While the SFX method is uniquely suited to probe ultrafast dynamics, serial synchrotron X-ray crystallography (SSX) can provide complementary data in the millisecond time domain [32,37,38]. Both SFX and SSX rely on the crystallization of the macromolecule of interest, and alternative methods are therefore required to study single particles in solution. Among these methods, cryogenic electron microscopy (cryo-EM) has become an important tool in structural biology since advances in detector technology and image processing brought about the “resolution revolution” [39]. Particles in solution can also be studied using X-ray scattering methods, including time-resolved wide-angle X-ray scattering (TR-WAXS) [25] and fluctuation X-ray scattering (FXS) [40]), albeit at limited spatial resolution. Finally, single particle imaging (SPI) at XFELs [41] provides an opportunity to capture structural information from macromolecules resistant to crystallization. A unique advantage compared to other single particle methods such as cryo-EM is that the use of the ultrafast X-ray pulses removes the need for cryogenic cooling of the samples. While SPI was first demonstrated on large particles such as the giant mimivirus [42], the technique has progressed in recent years, now allowing diffraction patterns to be collected from single proteins [43].

## Impact of high-repetition rate X-ray light sources

As discussed above, XFELs are able to capture the ultrafast chemistry and dynamics occurring within biological systems. However, the impact of these light sources has been limited due to the ~100 Hz rate at which the X-ray pulses are delivered at most facilities. Recently, a new generation of XFELs (the European XFEL and LCLS-II) has come online, providing X-ray pulses at a megahertz rate. The European XFEL, in operation since 2017, recently demonstrated the use of megahertz repetition rates for studying biological structure and dynamics using time-resolved SFX [44,45] and SPI [46]. Meanwhile, LCLS-II, which announced “first light” in 2023, will be upgraded to deliver hard X-rays (>5 keV) in the coming years (LCLS–II–HE) [47], making the facility suitable for structural biology experiments.

The ten-thousand-fold increase in the X-ray pulse rate of LCLS-II opens up new opportunities for studying biomolecular dynamics. For example, better sampling of the time axis in time-resolved pump-probe experiments allows for a more detailed mapping of the reaction coordinate. This will allow the rise and decay of reaction intermediates in heterogeneous samples to be captured in more detail (sometimes within a single crystal [48]), such that mixtures of intermediates can be modeled more accurately as a function of time. In addition, it is expected that a better temporal sampling will allow rare, short-lived events to be captured, without requiring prior information about reaction kinetics. Finally, we anticipate that the increased X-ray pulse rate will allow a wider parameter space and energy landscape of biological systems to be probed.

Large, comprehensive data sets will also benefit experiments with a limited signal-to-noise ratio by improving the precision of the measured structure factor amplitudes and scattering intensities through averaging. This will be particularly beneficial for experiments with a small differential signal (e.g., femtosecond pump-probe experiments) and experiments in which weak scattering signals are measured, including SPI, TR-WAXS, and FXS. In an SPI experiment, hundreds of thousands of snapshots have to be collected to reach near-atomic spatial resolution [49]. Hence, SPI, and other “data hungry” methods, will benefit significantly from an increased X-ray pulse repetition rate.

## Studying the structural dynamics of biomolecules using exascale computing

From the early days of computer simulation of biomolecules, developments in the capability of computers and simulation algorithms have helped transform our understanding of biomolecular structure, function, and dynamics. To appreciate how exascale computing can complement XFEL experiments for the next stage of development in this area, it helps to consider just what exascale computing can deliver.

The world’s first exascale supercomputer, Frontier, is able to deliver about 1.19 × 10^18^ floating point operations per second (flops) from its roughly 9400 compute nodes packed into 74 cabinets. Each node comprises 4 GPUs and 1 CPU (with 64 cores). The Frontier supercomputer not only has a smaller footprint relative to its predecessor Summit (peak performance 0.2 × 10^18^ flops) but it is also 3.3 times more energy efficient (as measured by flops/MW).

To better appreciate the capability of Frontier for biomolecular simulations, consider the simulation of Satellite Tobacco Mosaic Virus (STMV) in explicit water [50]. As Table 1 shows, the time to solution that required 256 nodes of a supercomputer in the year 2006 is now accessible with a single GPU on a standard workstation. The more capable GPUs in Frontier (or its predecessor, Summit) provide even better performance. Table 1 is meant only to provide a high-level guideline.

The important point is that just *a fraction of one node* of Frontier is already a very capable machine for biomolecular simulations.

### Ensemble computing for integrating with experiments

The Summit supercomputer enabled a record-breaking 10^9^-atom simulation [52]. Preliminary testing shows that with 512 nodes of Frontier, we can credibly simulate 8 × 10^9^ water molecules (Hagerty and Asthagiri, unpublished). But what is more interesting is the potential to study thousands of copies of a small (≾ 10^6^ atoms) system. The latter mode called *ensemble computing* not only enables enhanced sampling and scanning in parallel a large range of conditions of interest but is also of much interest in quantifying uncertainties [53]. The ensemble mode will be of high interest in integrating with experiments at XFELs. We briefly review promising methodological developments that are well-suited for ensemble computing.

### Simulations to enhance the interpretation of XFEL experiments

XFELs can provide a detailed picture of conformational changes that are critical to biomolecular function. These could be intrinsic conformational changes that occur at thermal energies or changes driven by external stimuli (e.g. changes in solution condition, (un)binding of ligands, T-jump, or E-fields). These are also the questions that simulations have sought to address over the past decades, allowing molecular dynamics simulations to be directly integrated with insights from XFEL experiments. For example, simulated crystalline electron densities were directly compared to experimental electron densities obtained from light-activated photosystem II crystals, providing unique insights into the mobility of the water networks inside the protein [33]. The ongoing development of several important molecular dynamics algorithms, described below, is expected to further enhance the integration of simulation with experimental data.

When experiments indicate the direction of change, for example, isomerization about a specific bond or the (un) binding along a well-defined pathway, those directions serve as natural *order parameters*. In this case, biased sampling techniques could prove particularly helpful [54–56]. When sampling is desired without applying any external biases, as would be required for obtaining kinetic properties, the highly scalable weighted ensemble technique presents itself as a compelling choice [57,58].

**Figure 3:**
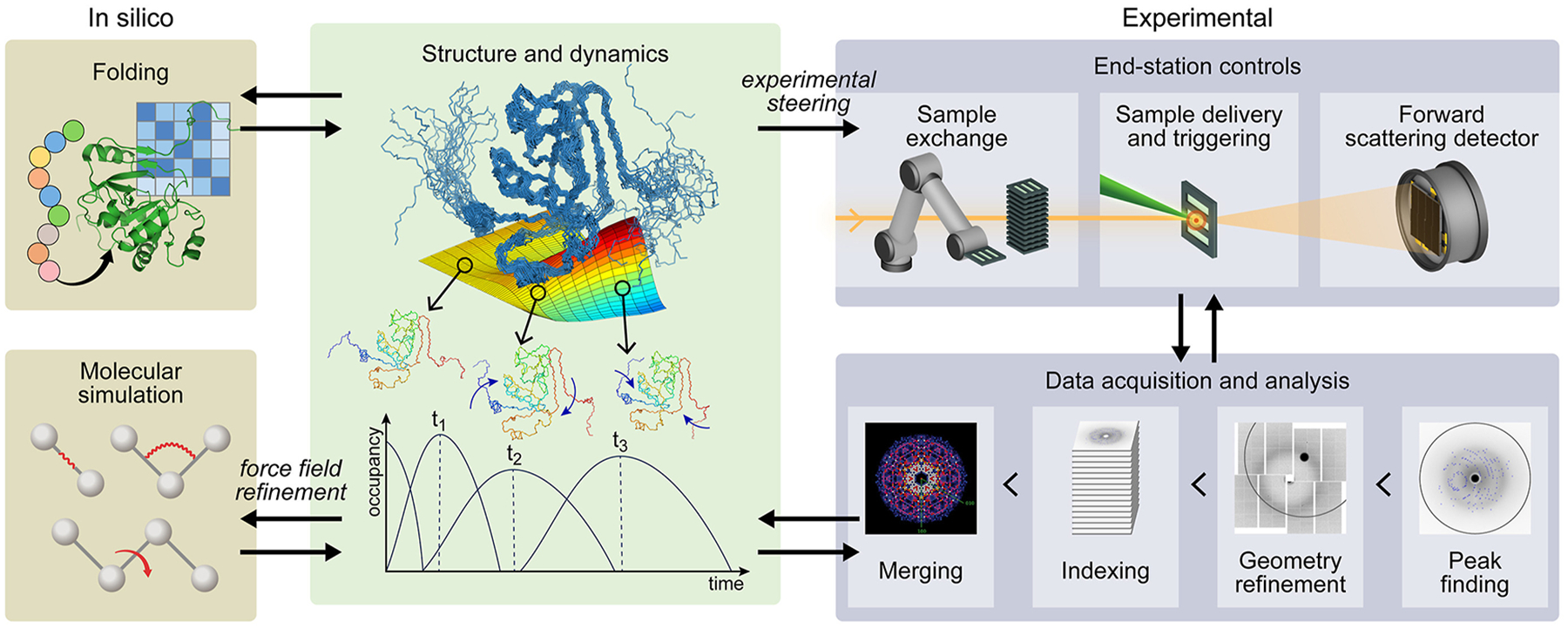
Molecular simulations (in silico) and time-resolved studies at XFELs (experimental) provide a comprehensive understanding of the structure and dynamics of macromolecules. Structural and dynamics information provided by simulations or experiments can be used to improve computational methods (e.g., through force field refinement) or help drive the experiment at the X-ray free-electron laser (experimental steering). The higher X-ray pulse rate of the new generation of XFELs will allow for an increased sampling of the time axis in a time-resolved experiment. In this way, the rise and decay of reaction intermediates and short-lived states can be captured more accurately.

When experiments make available a collection of conformational states, but the directions of change are not immediately obvious or several concerted (allosteric) motions are indicated, it becomes important to classify the various conformations and elucidate the mechanism of transitions among them. Markov State Models (MSMs) [59–63] based on a time-lagged independent component analysis (TICA) [64,65] are particularly compelling in this case. Based on a collection of vectors of user-specified order parameters, the TICA method finds those linear combinations, the basis, that de-correlate slowly and are thus most useful in describing the slowest relaxation processes, such as large-scale conformational changes of interest (Figure 3). Integrating adaptive sampling further enhances the efficiency and explanatory power of MSMs [66].

The comments above highlight the importance of order parameter(s). It is important to recognize that intuitively reasonable order parameters, such as the distance between a protein and a ligand, need not be the appropriate reaction coordinate for elucidating the kinetics [67]. To elucidate kinetics rigorously requires identifying the correct transition states, and this remains a challenge in high dimensions. An exciting recent development in this regard is machine-guided transition path sampling [68], wherein a neural network is trained to process a large collection of features, perhaps generate autonomously, to converge on a set of features that help locate the transition state. The final results can be interpreted using symbolic regression to derive chemically meaningful models.

Finally, developments in the molecular quasichemical theory (QCT) of solutions [69] now make it possible to obtain a comprehensive view of the thermodynamics of biomolecules in solution [70], treating the molecule holistically without relying on additivity assumptions that are often invoked in interpreting the thermodynamics of biomolecular transformations [71]. The QCT framework is rigorous, physically transparent, and designed to make good use of available data, be it from simulations or experiments. As such, the QCT framework is amenable to ensemble computing and can mesh with MSM or weighted sampling approaches to predict the thermodynamics of conformational substates.

## A vision for building an integrated research infrastructure coupling XFELs and exascale computing

The developments in XFEL and exascale computing are synergistic and can enable a comprehensive understanding of biomolecular dynamics. Figure 3 is our vision for integrating these two techniques. While experimental data is crucial for refining simulation parameters, integrating simulations into time-resolved data analysis can advance and improve the analysis and interpretation of experiments. We consider two specific possibilities, one in steering experiments using information from simulations and another in learning from experiments to improve simulations.

### Simulations to steer the XFEL experiments

Machine learning methods are increasingly capable of characterizing and calibrating XFEL hardware [72] by integrating simulation and experimental data (Figure 3). We envision that simulations, through providing a detailed kinetic model, can also predict the time delays at which we might find transition states and inform us about the triggering time delay (in applying external stimuli). A step in this direction is the use of a neural network to optimize experimental design [73]. In a similar vein, simulations could provide rich information about the macromolecule’s response to varying physical/chemical conditions (for example, ligand concentrations). This could provide a guide for identifying the most interesting samples to measure next, thereby informing the automated sample exchange.

### Learning from experiments to improve simulations

Above we tacitly assumed simulations are well-crafted and the force fields are adequate. The former is user-dependent, and the latter may not always be the case. For systems involving metal cofactors, we will need a quantum mechanical model for at least part of the metal-protein system. The physics of polarizability, often not included explicitly in standard force fields, and genuine many body effects could also play an important role. Modeling of nucleic acids is one area where some of these issues come to the fore [74]. However, the near one-to-one correspondence between XFEL experiments and simulations does open a way to use the knowledge from experiments to update the force fields for simulations.

### Challenges

**Figure 4:**
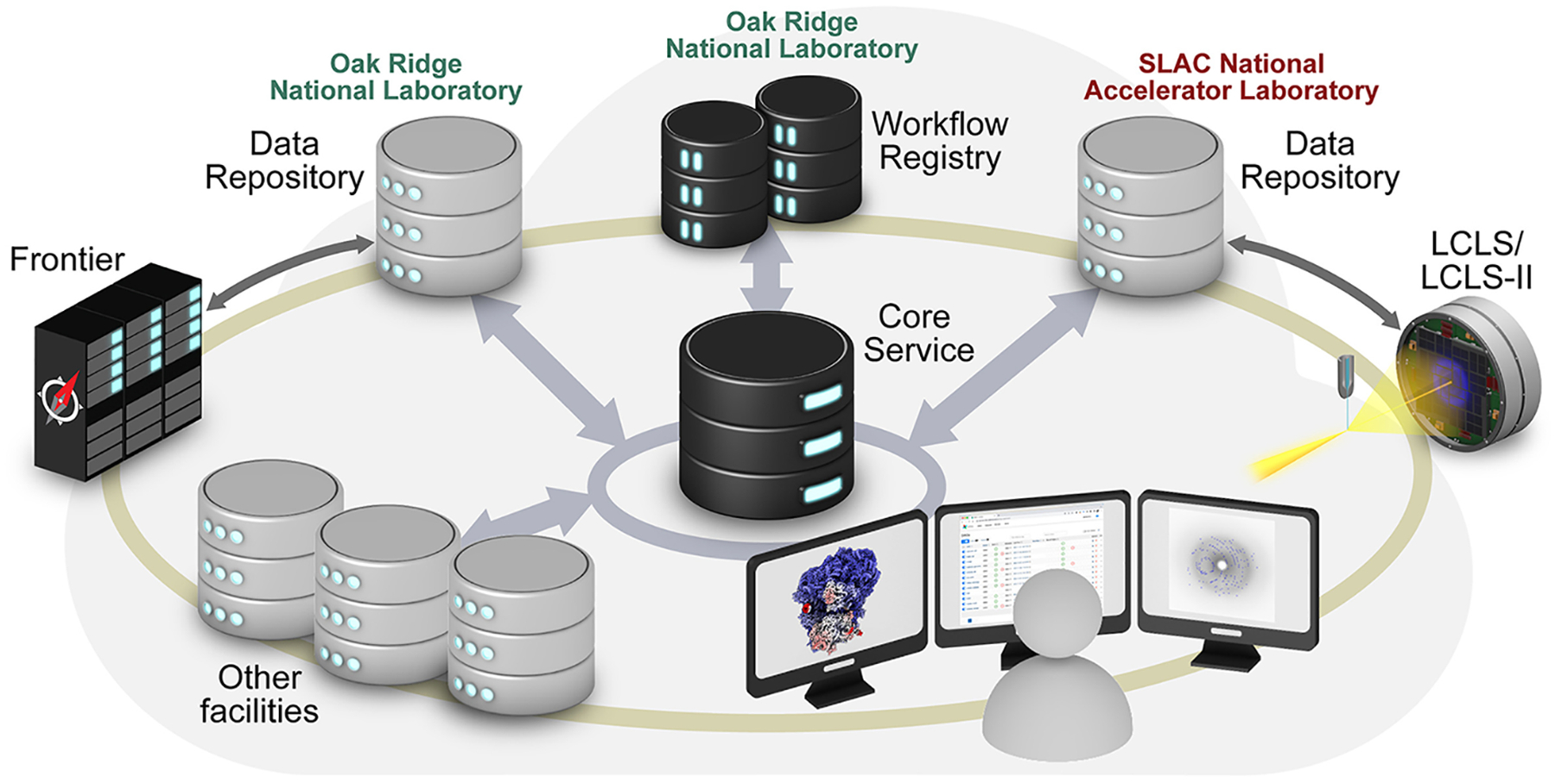
Data portal for high-throughput multimodal analysis. Structural and molecular dynamics data collected at the XFEL, such as LCLS or LCLS-II, will be registered in a central workflow registry to facilitate rapid collocation with data collected from other facilities and high-performance computing resources, such as the Frontier exascale supercomputer. These Integrated Research Infrastructures (IRI) will enable data processing, reprocessing, and large-scale multimodal studies.

We have thus far sketched the potential for coupling XFELs and exascale computing, and the tools and techniques that would be of most interest. We briefly highlight key challenges. While being US-centered, it is informative to consider the specifications for the plans of the US Department of Energy to build an integrated research infrastructure between its major experimental facilities, including XFELs, and the leadership computing facilities (Figure 4): the total aggregate and peak compute performance should be on the order of exascale, file systems must support high-throughput, concurrent data writing and reading from many sources at once, centralized data storage facilities should have capacities up to exabytes and networking bandwidths should be on the order of terabytes per second. The current standard for the DOE ESnet transmission rate is about 50 gigabytes per second, illustrating the importance of effective data compression and motivating the push to higher bandwidths across the network.

We should note that in addition to these infrastructure challenges, the many-fold increase in data generation capacity of LCLS-II also calls for algorithmic developments leveraging the state of the art in computer vision and structure generation from diffraction patterns arising from a large number of randomly oriented samples. The Exascale Computing Project within the DOE developed a wide range of such software tools and libraries, including the exaFEL suite of codes [75]. In addition, artificial intelligence and machine learning techniques are being developed to enable GPU parallel computing [76,77] and tackle data compression [78,79], so that actionable information can be extracted quickly and accurately from the deluge of data back to steer the experiment. Finally, to truly provide scientists with a seamless integration of data and algorithms that informs them on demand, it will be crucial to track data and processing workflows through delocalized registries across all facilities.

## Conclusions

Being able to obtain time-resolved pictures of molecular transformations from experiments at the XFEL and simultaneously study the same in computer simulations opens broad possibilities for a deeper understanding of biomolecular structure, dynamics, and function at ambient temperatures. Technical challenges to integrate experimental and computational facilities separated by a continent are being addressed, and those developments will also favorably impact other areas of science and technology. We are at the threshold of a new age that fully integrates experiments and computing at a level that was not previously possible.

## Figures and Tables

**Table 1:** Approximate times to obtain 1 ns of dynamics with a 1 fs time-step for the STMV model system comprising about 1.07 × 10^6^ atoms. Throughout, we use 7 cores per GPU. Ref. [50] is the original study of the STMV model using 256 Altix nodes at the National Center for Supercomputing Applications (NCSA). The desktop workstation (built year 2022) is equipped with the NVIDIA RTX 6000 GPU. Frontier nodes have the AMD MI250X GPUs. Simulations were performed with the NAMD [51] (ver. 3.0), with the runs on Frontier in the so-called GPU-resident mode.

Machine	hrs/ns
Ref. 50 (year 2006)	21.6
Workstation (with GPU)	16.8
Frontier 1 GPU	4.32
Frontier 2 GPU	2.40
Frontier 4 GPU	1.20

## Data Availability

Data will be made available on request.
